# A Prospective Clinical Audit of Sterile Surgical Glove-Donning Practices Among Young Surgeons at a Tertiary Care Hospital in Sudan

**DOI:** 10.7759/cureus.68493

**Published:** 2024-09-02

**Authors:** Alsadig Suliman, Ali Mohammed Ibrahim, Siddig Ali, Hiba Suliman

**Affiliations:** 1 General Surgery, Sudan Medical Specialization Board, Khartoum, SDN; 2 General Surgery, Wad Al-Naeem Hospital, Wad Madani, SDN; 3 General Surgery, Wad Medani College of Medical Sciences and Technology, Wad Madani, SDN

**Keywords:** compliance improvement, donning sterile surgical gloves, infection control guidelines, surgical asepsis, surgical audit, surgical site infections (ssi), who guidelines

## Abstract

Background

Adherence to aseptic protocols and proper sterilization is critical for optimal patient recovery post-surgery. The standard procedure for donning sterile surgical gloves helps manage infection risk and maintain surgical site cleanliness by following aseptic principles. This study evaluates adherence to these protocols among young surgeons at a tertiary care hospital in Sudan.

Methods

This prospective audit included 42 young residents and house officers at a tertiary care hospital in Sudan, following ethical clearance. Compliance with sterile surgical glove-donning practices was assessed using the World Health Organization (WHO) pre- and post-intervention criteria. Participants were observed in the operating room without prior notice. The intervention comprised a video presentation and a live demonstration.

Results

Pre-intervention adherence to standard criteria for donning sterile surgical gloves was 73.4% (n= 31). This rate increased significantly to 91.4% (n= 38) following the intervention and showed marked improvement.

Conclusion

The audit demonstrated a significant improvement in adherence to donning sterile surgical gloves practices after the intervention. Implementing WHO guidelines effectively enhances compliance, reduces infection risks, and increases awareness of aseptic practices.

## Introduction

Sterile surgical glove-donning practices are crucial for maintaining aseptic conditions during surgical procedures. Proper glove use is essential to prevent surgical site infections (SSIs) [1]. SSIs are a significant concern within the spectrum of hospital-acquired infections (HAIs), with a pronounced impact on morbidity, mortality, and healthcare costs, particularly in developing countries [2]. SSIs are defined as infections occurring at the incision site or in deep tissues within 30 days post-surgery, or up to a year if an implant is involved [3]. In developed countries, SSIs impact around 5-10% of hospitalized patients, highlighting how common they are and the need for strict infection control measures [4]. Despite being the most common type of nosocomial infection among post-operative patients, SSIs are also among the most preventable HAIs [4,5].

In resource-limited settings such as Sudan, maintaining high infection control standards is particularly critical [6]. Recent studies highlight the importance of strict adherence to sterile techniques. For instance, the World Health Organization (WHO) guidelines on SSI prevention highlight meticulous hand hygiene (HH) and proper donning of sterile gloves as key measures [7]. The Centers for Disease Control and Prevention (CDC) also provides comprehensive guidelines for proper glove-donning and infection control practices [8]. Additionally, recent studies show that consistent use of proper glove-donning procedures significantly reduces SSIs [9].

This audit was initiated in response to observed variations and potential inconsistencies in sterile surgical glove-donning techniques among young surgeons at a tertiary care hospital in Sudan. It evaluates glove-donning practices in the elective general surgery operating room (OR) guided by WHO guidelines. As the first prospective audit of its kind in Sudan, it aims to both educate and assess participants in the General and Laparoscopic Surgery department. The objective is to address these concerns and improve surgical outcomes by evaluating and enhancing glove-donning practices among trainees. A multidisciplinary team, including senior surgeons, infection control specialists, nursing staff, and hospital administration, has been convened for this purpose. We hypothesize that the fast-paced OR environment, limited training duration, and a lack of clear objectives for each year of training are the major problems of training and present challenges for general surgery residents and house officers in mastering sterile glove-donning skills [10-12].

## Materials and methods

Following approval from the Ethical Review Board of the Hospital (approval number: 2023/ERB/005), granted in August 2023, this two-week audit was conducted in the elective OR of the General and Laparoscopic Surgery department from August 17, 2023, to August 31, 2023. Observations were carried out on two elective surgery days each week. A total of 42 surgical residents and house officers participated in the study. An extensive literature review informed the research plan and design.

Pre-intervention observation

In the third week of August, young surgeons were observed donning sterile surgical gloves before elective surgeries on two separate days. Observations were compared against the WHO's standard guidelines for glove donning by a team of six auditors. We assessed all young surgeons based on the evaluation criteria established by the WHO [1,13]. Compliance was evaluated using 15 criteria, as detailed in the table we used for assessment (Table 1). Each correct response (yes) was assigned a score of 1, while each incorrect response (no) received a score of 0. Compliance scores for each criterion were totaled across participants, and percentages were calculated to determine overall compliance for each criterion.

**Table 1:** WHO guidelines on donning sterile surgical gloves.

Criteria	Description	Response	
1	Perform hand hygiene using an alcohol-based hand sanitizer or soap and water before donning sterile gloves.	Yes	No
2	Choose the proper glove size to ensure a snug fit without compromising dexterity.	Yes	No
3	Verify that the glove package is intact and has not been compromised.	Yes	No
4	Open the glove package carefully by peeling back the outer layer to expose the gloves, and avoid touching the inner surface of the package.	Yes	No
5	Place the glove package on a sterile, dry surface, ensuring that the gloves do not come into contact with any non-sterile object.	Yes	No
6	Identify the right and left gloves. Ensure you start with the glove for the dominant hand.	Yes	No
7	Using the thumb and index finger of the dominant hand and grasp the inside of the glove at the cuff, without touching the outer surface.	Yes	No
8	Slide the dominant hand into the glove in a single smooth motion, keeping the glove cuff at wrist level, and avoid any contact with the outside of the glove or surrounding surfaces.	Yes	No
9	With the gloved hand, pick up the second glove by sliding the fingers under the cuff.	Yes	No
10	In a single movement, slip the second glove into the ungloved hand, ensuring that the glove cuff remains at the wrist level.	Yes	No
11	Adjust both gloves to ensure a proper fit, without touching non-sterile surfaces or other parts of the gloves that are not sterile.	Yes	No
12	Verify that gloves cover the wrists completely and that there are no gaps between the glove and the wrist.	Yes	No
13	Keep hands above waist level and below shoulder level without any contact with non-sterile areas.	Yes	No
14	Ensure that the gloved hands only touch sterile equipment or areas that have been disinfected.	Yes	No
15	If the gloves become contaminated, remove and dispose of them properly, perform hand hygiene, and don a new pair of gloves.	Yes	No

The intervention

Following the initial two weeks of observation and data collection, participants were introduced to an educational intervention. This included a presentation featuring a live demonstration and a video on proper sterile surgical glove-donning techniques according to WHO guidelines. Additionally, a consultant surgeon provided an individual demonstration in the OR for the residents and house officers. An illustrated instruction manual on the standard of donning sterile surgical gloves were also placed in the OR.

Post-intervention assessment 

In the second phase of the audit, conducted during the final week of August, all participants were reassessed for their compliance with the standard criteria for donning sterile surgical gloves. Each correctly performed step was awarded 1 point, and all 15 steps were individually evaluated. Participants received a total score out of 15 (1 step = 1 point), and compliance scores were calculated both individually and collectively to determine the average improvement. For each participant, compliance with these 15 criteria was calculated, and the scores for each criterion were summed across all participants. Data analysis was performed using the Statistical Package for Social Sciences (SPSS) version 26.0 (IBM Corp., Armonk, NY), with frequencies and mean values calculated to assess individual and group compliance rates before and after the intervention. Participants also provided feedback on the effectiveness of the intervention methods for learning the standard practice of donning sterile surgical gloves.

## Results

**Figure 1:**
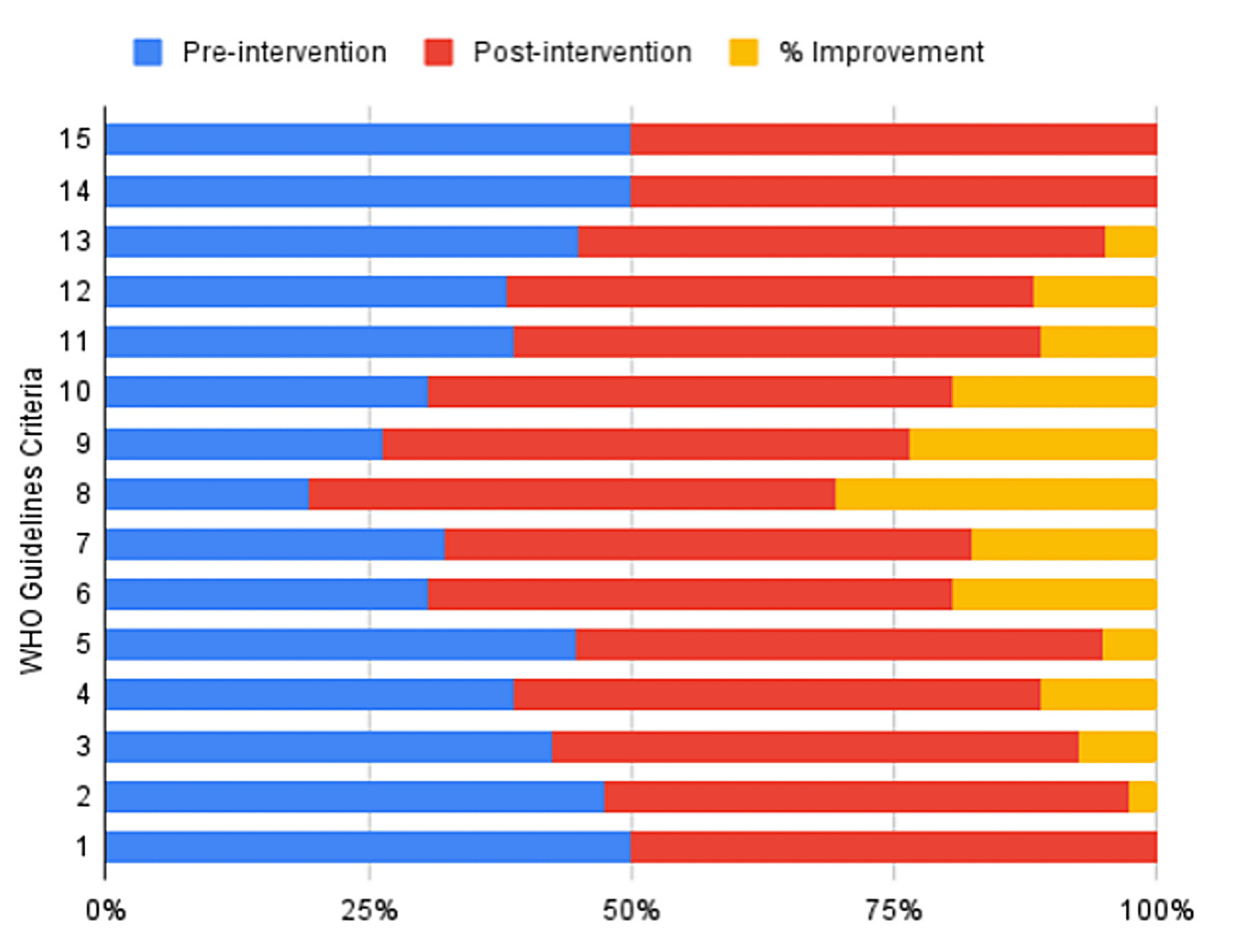
Percentage of compliance for pre-evaluation, post-evaluation, and improvement across various steps in donning sterile surgical gloves.

**Figure 2:**
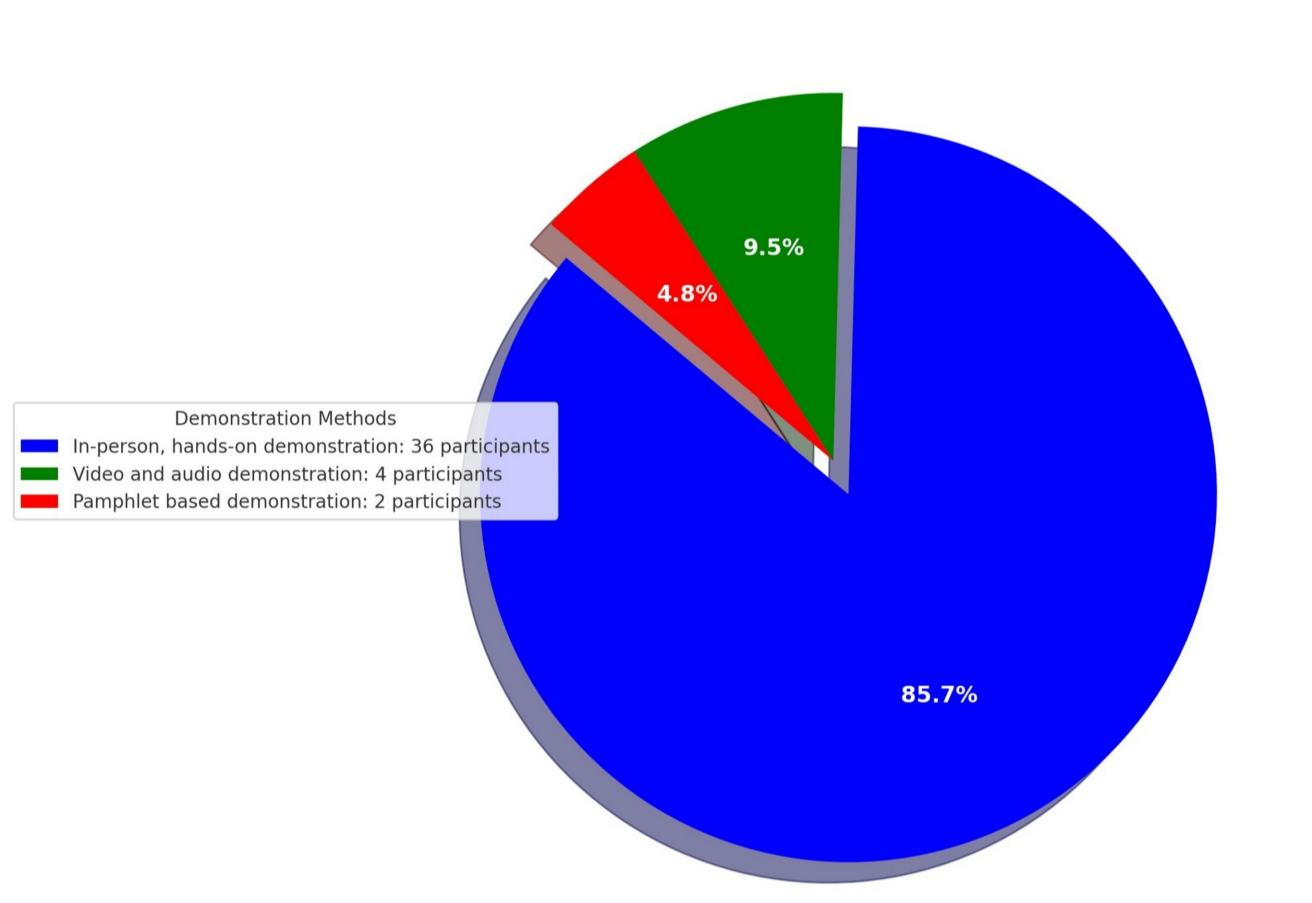
Participant's preferred demonstration method.

The study monitored 42 surgical residents and house officers (29 males, 13 females) to assess their awareness and application of sterile surgical glove-donning practices. The most significant improvement was observed in criterion 8: "Slide the dominant hand into the glove in a single smooth motion," with compliance increasing from 35% (N=14) pre-intervention to 90% (N=38), a difference of 55%. Following the intervention, adherence to criteria 6, 9, and 10 also improved significantly. Before the intervention, compliance rates for these criteria were 55% (N=23), 45% (N=19), and 55% (N=23), respectively. After the intervention, these rates increased to 90% (N=38), 85% (N=36), and 90% (N=38), respectively. In comparison, the OR health personnel at the hospital achieved a compliance rate of 90% for these criteria, while the young surgeons in our ward exhibited full compliance at 100%. Overall, compliance rates improved from 73.4% (N=31) to 93% (N=39), representing a mean improvement of 19.7% (Table 2). Criteria 1, 14, and 15 showed no change in compliance rates, as these steps were already being followed correctly by all participants (Figure 1). Feedback indicated that 85.7% (N=36) of participants found the "In-person, hands-on demonstration" to be the most effective method for learning proper sterile surgical glove-donning practices (Figure 2).

**Table 2:** Pre-intervention and post-intervention compliance percentage improvement for various criteria.

Criteria	Compliance/adherence rates				Improvement%
	Pre-intervention		Post-intervention		
	%	N	%	N	
1	100	42	100	42	-
2	90	38	95	40	5
3	85	36	100	42	15
4	70	29	90	38	20
5	85	36	95	40	10
6	55	23	90	38	35
7	55	23	85	36	30
8	35	14	90	38	55
9	45	19	85	36	40
10	55	23	90	38	35
11	70	29	90	38	20
12	65	27	85	36	20
13	90	38	100	42	10
14	100	42	100	42	-
15	100	42	100	42	-
Mean compliance	73.4	39	93	31	19.7

## Discussion

The proper donning of sterile surgical gloves is crucial for maintaining asepsis and promoting effective wound healing. Achieving proficiency in this skill requires both proper instruction and regular practice [1]. Our study demonstrated a mean improvement of 19.7% in compliance with sterile surgical glove-donning practices. Initially, we identified a significant lack of awareness regarding critical steps in the glove-donning process. However, after implementing our targeted intervention, we observed a substantial improvement in this area.

Comparing our findings with other studies, our results align with a study conducted in Pakistan, which reported a mean improvement of 18.5% in sterile glove-donning practice and a post-intervention compliance rate of 90.71% [1]. Additionally, a study from Sudan evaluating hand scrub compliance using similar methods found that continuous education through videos, positive feedback, and guidance significantly improved compliance from 63% pre-intervention to 90.33% post-intervention [14]. A review of 46 studies conducted worldwide concluded that after intervention, HH compliance improved, ranging from 1% to 66%, with a mean net improvement of 26% [1].

Reviewing existing literature and our findings, it is evident that increasing compliance among healthcare workers requires regular audits and educational interventions, such as pictorial guides, video demonstrations, and individual training [15,16]. Similarly, an Indian study, which included a three-month baseline and a four-month intervention period, observed a peak HH compliance rate of 90% at the two-year follow-up, though it declined to 82% by the three-year mark, highlighting the need for ongoing audits [16,17]. Improved compliance in sterile glove donning can reduce SSIs and enhance patient outcomes. Kasula et al. stressed the importance of teaching and reinforcing surgical glove-donning skills early in medical training. To ensure sustained compliance, healthcare institutions should invest in regular training sessions, visual aids, and routine audits [18]. This study highlights the effectiveness of targeted educational efforts in significantly enhancing procedural skills and understanding of aseptic principles.

Despite these promising results, our study had several limitations. Factors such as lack of motivation and demanding duty hours contributed to decreased compliance. The single-center design and the majority of participants being from the General Surgery department, along with the small sample size, limit the generalizability of our results. Additionally, reliance on direct observation may have introduced bias, as participants might alter their behavior when being observed. Variations in individual compliance due to differences in background, experience, religion, and personal habits were not controlled for and might have influenced adherence rates.

Future research should focus on multicenter studies with larger sample sizes to confirm these findings and assess long-term sustainability. Exploring different methods of intervention and their impact on patient outcomes would provide a more comprehensive understanding of how to maintain high compliance rates [19].

## Conclusions

This clinical audit highlights the critical role of ongoing assessments and training in enhancing patient safety. It underscores the importance of adhering to proper sterile surgical glove-donning practices to reduce SSI rates associated with HAIs. The significant improvements observed post-intervention confirm the effectiveness of targeted training in increasing adherence to WHO recommendations regarding sterile surgical glove-donning practices. Regular evaluations and continuous education are essential to foster better compliance with protocols. In conclusion, while our study shows a positive trend toward better practices, regular audits are crucial for sustaining improvements in sterile surgical glove-donning compliance. Implementing these strategies can ultimately enhance patient safety and healthcare quality.
